# 

**Published:** 1913-09-15

**Authors:** 


### ANNOUNCEMENTS.

Ohio State Dental Society. The local committee of arrangements has already begun to boost for the next meeting of this Society. The next meeting will be held in Toledo, and it goes without saying that a strenuous attempt will be made to break all previous records in the matter of attendance as well as in the attractiveness of the program. It is intended that the best literary talent that the country affords only will be invited to participate in the program and the clinic will be limited to fifteen and each of these will be the best obtainable. The committee has a surprise in store for all who attend that will make it well worth your while to begin to plan for this meeting now so that you will not be disappointed.

Examinations for Dentists in the Army, will be held October 13th at the Columbus Barracks, and at other Army Stations. Send to the Surgeon General, U. S. Army, Washington, D. C. for the application blanks and other information as to places where the examinations can be had. Only graduates between 21 and 27 years of age are eligible The pay is $150 per month with certain requisites, and a three years contract is made.

---

THE SIXTH INTERNATIONAL DENTAL CONGRESS, is being organized to be held August 8th in London, England, under the auspices of the British Dental Association, and the patronage of his Majesty, King George V., J. Howard Mummery will be the Presiding officer, and Norman G. Bennett, the Secretary. Any ethical practitioner of this country can attend and become a member.

---

THE FORSYTH DENTAL INFIRMARY OF BOSTON, will open for work among the poor children of Boston about October first. This is one of the finest charities that has ever been dedicated to suffering humanity. A magnificent marble building with a most complete outfit for taking care of the 200,000 poor school children of Boston and its near cities. The staff will consist of competent experts in various lines assisted by a corps of paid operators and senior students from Harvard and Tuffts Colleges. There are a number of positions open to graduates at $1000 per year for full time work and shorter time with correspondingly less pay.

For full particulars address the DIRECTOR DR. HAROLD W. CROSS, 149 Tremont St., Boston, Mass.

---

THE NORTHERN INDIANA DENTAL SOCIETY will hold its annual meeting this year at Gary, September 23rd to 25th.

W. L. MEYERS, *Secy.*

